# Use of Age-Stage, Two-Sex Life Table to Compare the Fitness of *Bactrocera dorsalis* (Diptera: Tephritidae) on Northern and Southern Host Fruits in China

**DOI:** 10.3390/insects13030258

**Published:** 2022-03-04

**Authors:** Yanfei Zhu, Fangjian Qi, Xiumei Tan, Tong Zhang, Ziwen Teng, Yinjun Fan, Fanghao Wan, Hongxu Zhou

**Affiliations:** 1 Key Laboratory of Integrated Crop Pest Management of Shandong Province, China-Australia Joint Institute of Agricultural and Environmental Health, College of Plant Health & Medicine, Qingdao Agricultural University, Qingdao 266109, China; yanfeizhu2021@163.com (Y.Z.); q13054849269@163.com (F.Q.); 199601018@qau.edu.cn (X.T.); zero0852580@163.com (T.Z.); tzwbat@126.com (Z.T.); fanyinjun89@126.com (Y.F.); 2 Agricultural Genomics Institute at Shenzhen, Chinese Academy of Agricultural Sciences, Shenzhen 518120, China

**Keywords:** *Bactrocera dorsalis*, age-stage two-sex life table, host fruits, fitness, population projection

## Abstract

**Simple Summary:**

Influenced by global climate, trade, and transportation factors, *Bactrocera dorsalis* (Hendel) has gradually spread from southern regions to Beijing, Hebei, and other northern areas in China. In order to evaluate the risk of damage of *B. dorsalis* to the dominant northern fruits, an age-stage two-sex life table was used to study the fitness of *B. dorsalis* for peaches and apples, with oranges as the control. Our results showed that the pest can cause continuous damage on northern fruit hosts, with the damage degree being basically the same on peaches as on the southern fruit of oranges. The *B. dorsalis* population on peaches increased by 12,112.1 times 90 days after oviposition, easily causing great damage. Additionally, the population increased by 4311 times on apples, which may also become a potentially new host of *B. dorsalis* in northern China, though with relatively lower fitness. This research lays a foundation for monitoring and the formulation of efficient control strategies for *B. dorsalis*.

**Abstract:**

*Bactrocera dorsalis* (Hendel), as a quarantine pest in many countries and regions, has shown a trend of northward diffusion in the past century in China. In order to determine whether *B. dorsalis* will cause great harm to the dominant northern fruits, the age-stage two-sex life tables of peaches and apples were constructed, with oranges as the control. The results showed that the developmental rate, intrinsic rate of increase (*r*), and finite rate of increase (*λ*) on oranges and peaches were significantly greater than on apples. Additionally, the prediction of population growth 90 days after oviposition revealed that the whole population on oranges and peaches increased by 13,667.3 and 12,112.1 times, respectively, indicating that *B. dorsalis* is very likely to endanger peach orchards. The population increased on apples by 4311 times, though this is lower than that on oranges and peaches. Overall, peaches with high fitness similar to oranges are very suitable as a host for *B. dorsalis* and are likely to become a new favorable host, while apples may also become a potentially new host, though with lower fitness. Therefore, the most pressing solutions to take are population monitoring, comprehensive prevention, and control in the case of any potential large-scale outbreak of *B. dorsalis* in northern China.

## 1. Introduction

*Bactrocera dorsalis* (Hendel) (Diptera: Tephritidae), one of the most destructive pests in the Asia-Pacific region [1], can harm more than 250 kinds of fruits and vegetables such as guava, mango, peach, and apple [2]. In 1997, *B. dorsalis* caused economic losses of $1.26 billion in Taiwan, China [3]. *Bactrocera dorsalis* has been listed among quarantine targets, and strict quarantine measures on fruit import and export have been implemented in many countries and regions [4].

With the influence of the global climate, transportation activities and other factors, the spread of *B. dorsalis* has gradually accelerated [5]. It was only reported in southern China in the 20th century [6], but in past decades, *B. dorsalis* adults have been gradually monitored in Henan, Shaanxi, Beijing, and Hebei in northern China [7,8,9,10], which are the important fruit producing areas of peach, apple, pear, and other fruits (https://data.stats.gov.cn/easyquery.htm?cn=C01&zb=A0D0F&sj=2020, accessed on 8 March 2021). Whether the areas would be exposed to potential damage caused by *B. dorsalis* still remains unclear.

Clarifying the occurrence dynamics of invasive pests in different environments and their potential population growth in new habitats will help to formulate corresponding prevention and control strategies [11]. Life tables are an important tool for monitoring field dynamics and studying population ecology [12], and are a vital means to evaluate the risk of damage from invasive pests [13]. Chi and Liu [14] and Chi [15], based on traditional life tables, proposed an age-stage two-sex life table in which population prediction plays a crucial role in pest control [12,16]. At present, the age-stage two-sex life table has widely been used in the study of some mites such as *Luciaphorus perniciosus* Rack (Acari: Pygmephoridae) [17] and pest insects including *Bemisia tabaci* (Gennadius) (Hemiptera: Aleyrodidae) [18] and *B. dorsalis* [1,19]. Previous studies on *B. dorsalis* hosts in China have mainly focused on tropical and subtropical fruits including guava, banana, papaya, pitaya, sweet orange, pomelo, wax apple, and mango in southern areas [1,19], and the situation of *B. dorsalis* on northern host fruits is still unclear.

In terms of the northward spread of *B. dorsalis* in recent years, according to the first-generation damage of *B. dorsalis* on oranges, peaches, and apples (Figure S1), it is a pressing issue to determine whether *B. dorsalis* will cause continuous damage to the dominant fruits in northern China. In this study, second-generation of *B. dorsalis* fed with oranges (a southern fruit), peaches, and apples (two dominant northern fruits) were taken to construct age-stage two-sex life tables, and their growth, development, survival, and reproduction were analyzed to evaluate the fitness of different host fruits. The growth trend of *B. dorsalis* population was also predicted, with the purpose of offering a theoretical support for population monitoring and integrated prevention and control of *B. dorsalis* in northern China.

## 2. Materials and Methods

### 2.1. Insects

*Bactrocera dorsalis* was collected from the research group at the College of Plant Protection, South China Agricultural University, and has been raised indoors continuously for many generations. After being transferred to our laboratory, *B. dorsalis* was raised with artificial feeding at 26 ± 1 °C, 65 ± 5% RH, and 16:8 (L: D) h in an artificial climate room. The feeding method referred to Cheng et al. [20] and was slightly adjusted (larval diet containing 150 g corn flour, 0.6 g sodium benzoate, 30 g yeast, 30 g sucrose, 30 g paper towel, 1.2 mL hydrochloric acid, and 300 mL water; adult diet consisting of water, yeast hydrolysate, and sugar). During this period, the development rate, fecundity, and longevity of the experimental insects were stable.

### 2.2. Host Fruits

The oranges (*Citrus reticulata* Blanco), peaches (*Amygdalus persica* L.), and apples (*Malus pumila* Mill., Red Fuji) for the experiment, purchased from Chengyang Wholesale Market in Qingdao, Shandong Province, China, were all fresh and free of pests and diseases. The products in the fruit market were in compliance with the regulations of the pesticide residue monitoring and management department. In addition, we washed and soaked the fruit in sterile water for 2 h after purchase to prevent pesticide residue.

### 2.3. Life Table Study 

Eggs hatched from the first-generation *B. dorsalis* (100 males and 100 females) fed with oranges, peaches, and apples were collected in 220 mL perforated paper cups (500 holes pierced with an insect needle number five) for 24 h; then, 15 eggs were placed with a brush into a Petri dish (60 mm in diameter) containing corresponding host fruits with six replicates for each treatment, for a total of 90 eggs. The hatched larvae were numbered in sequence and transferred to a new Petri dish, and their survival numbers were recorded daily. When mature, the larvae were placed in sandy soil at 3 cm depth and 65 ± 5% RH to pupate. Twenty-four hours after pupation, the pupae were taken out to count their numbers and to be weighed after gently sweeping the soil off the shell with a writing brush. Five pupae taken randomly for each treatment were weighed with an electronic balance (AB204-N, Mettler Toledo, Boston, MA, USA); this was repeated 10 times. Finally, the newly-emerging adult pairs were placed in adult cups composed of one 200 mL and one 340 mL plastic cup for daily observation of their survival and reproduction. If more males than females emerged on a given day or if any females died during the experiment, the insufficient females were supplemented from the corresponding population for pairing, but their survival was not recorded, and vice versa.

### 2.4. Life Table Analysis

The age-stage two-sex life table was conducted [14,15] using the TWOSEX-MSChart program [21]. Population parameters include the age-stage specific survival rate (*s_xj_*, *x* = age and *j* = stage), age-specific survival rate (*l_x_*), age-stage specific fecundity (*f_xj_*), age-specific fecundity (*m_x_*), age-stage life expectancy (*e_xj_*), reproductive value (*v_xj_*), and life table parameters, including net reproductive rate (*R*_0_), intrinsic rate of increase (*r*), finite rate of increase (*λ*), and mean generation time (*T*) [14,15].

In the age-stage, two-sex life table [14], *m_x_* and *l_x_* are calculated as:

$$m_{x}=\frac{\sum_{j=1}^{k}s_{xj}f_{xj}}{\sum_{j=1}^{k}s_{xj}}$$

$$l_{x}=\overset{k}{\underset{j=1}{\sum }}s_{xj}$$

where *k* is the number of stages, *s_xj_* is the probability that a newborn will survive and grow to age *x* and stage *j*, and *f_xj_* is the mean number of offspring produced by a female at age *x*. The net reproductive rate (*R*_0_) is calculated as:

$$R_{0}=\overset{\infty }{\underset{x=0}{\sum }}l_{x}m_{x}$$

The intrinsic rate of increase (*r*) is determined using the Euler–Lotka equation with age indexed from 0 [22]:

$$\;\overset{\infty }{\underset{x=0}{\sum }}e^{-r\left(x+1\right)}l_{x}m_{x}=1$$

The finite rate of increase (*λ*) is calculated as $\lambda =e^{r}$, and the mean generation time (*T*) is defined as the length of time that a population needs to increase its size *R*_0_-fold ($e^{rT}=R_{0}$ or $\lambda^{T}=R_{0}$) at a stable age-stage distribution. The calculation formula is as follows:

$$T=\frac{\ln R_{0}}{r}$$

*e_xj_* refers to the length of time that an individual of age *x* and stage *j* is expected to live, which is calculated according to the formula described by Chi and Su [23]:

$$e_{xj}=\overset{\infty }{\underset{i=x}{\sum }}\overset{k}{\underset{y=j}{\sum }}s_{iy}^{\prime }$$

where $s_{iy}^{\prime }$ is the probability that an individual of age *x* and stage *j* will survive to age *i* and stage *y*, assuming that $s_{xj}^{\prime }=1$ [23]. The reproductive value (*v_xj_*) refers to the contribution of individuals at age *x* and stage *j* to the future population, and the calculation formula is [24]:

$$v_{xj}=\frac{e^{r\left(x+1\right)}}{s_{xj}}\overset{\infty }{\underset{i=x}{\sum }}e^{-r\left(i+1\right)}\overset{k}{\underset{y=j}{\sum }}s_{iy}^{\prime }f_{iy}$$

The standard errors of all life table parameters, including hatching rate, pupation and eclosion rate, pupal weight, *r*, *λ*, *R*_0_, *T*, egg duration, larval duration, pupal duration, pre-adult survival rate, adult duration, female adult longevity, male adult longevity, total longevity, oviposition days, oviposition period, fecundity, TPOP (total pre-oviposition period from egg to first oviposition), proportion of female adults, and proportion of male adults, were estimated using the bootstrap procedure with 100,000 resampling. The paired bootstrap test was used to detect differences among treatments according to the confidence interval of differences [25].

### 2.5. Population Projection

The TIMING-MSChart program [26] was used to predict the population growth rate and the structure of each age-stage of *B. dorsalis* based on data such as hatching rate, survival rate, fecundity, and the duration of each stage [14,16].

## 3. Results

### 3.1. Life History Statistics of B. dorsalis on Three Host Fruits

Table 1 shows that there were no significant differences in the rates of hatching, pupation, and eclosion on the three host fruits. Meanwhile, the pupal weight of *B. dorsalis* was significantly different among the three hosts, being the heaviest on oranges (16.33 mg), where it was significantly heavier than on peaches (15.06 mg, *P* < 0.0001) and apples (14.38 mg, *P* < 0.0001), with a significant difference between the latter two fruits (*P* = 0.0175).

Table 2 indicates the developmental stages of *B. dorsalis* fed on different host fruits. As is shown, there was no significant difference in both larval duration and pre-adult survival rate in *B. dorsalis* on the three hosts. Furthermore, no significant difference was shown in egg and adult durations on peaches and oranges, but the pupal stage on the former was significantly shorter than on the latter (*P* = 0.0005). However, the egg duration of *B. dorsalis* fed on apples was the longest, significantly longer than on oranges (*P* = 0.0003) and peaches (*P* = 0.0107); and the adult duration on apples (65.35 day) was also significantly shorter than on oranges (75.42 day, *P* = 0.0063).

With regard to the reproduction of *B. dorsalis*, there was no significant difference in oviposition days and oviposition period on oranges (66.00 day, *P* = 0.0032) and peaches (63.86 day, *P* = 0.0069), both of which were significantly longer than on apples (52.50, *P* = 0.0032, *P* = 0.0069, respectively). As far as TPOP and fecundity are concerned, there were significant differences among the three hosts, among which the TPOP on apples (35.85 day) was the longest, significantly longer than on peaches (30.14 day, *P* < 0.0001) and oranges (29.30 day, *P* < 0.0001), and the fecundity on oranges (1157.33) was the highest, followed by peaches (910.57, *P* = 0.0009) and apples (723.21, *P* < 0.0001) (Table 2).

In terms of longevity (Table 2), there were no significant differences in female adults and total longevity of *B. dorsalis* among the three host fruits. However, the longevity of male adults was the longest on oranges (68.58 day), significantly higher than that on peaches (59.82 day, *P* = 0.0451) and apples (53.41 day, *P* = 0.0006). Furthermore, for *B. dorsalis* fed on the same fruit hosts, the longevity of male adults was significantly shorter than female adults on oranges (*P* = 0.0007), peaches (*P* < 0.0001), and apples (*P* = 0.00003).

As for the proportion of male-to-female adults, there were no significant differences in the proportion of female adults (*N_f_*/*N*) and male adults (*N_m_*/*N*) on the three different fruit hosts.

### 3.2. Life Table Parameters of B. dorsalis on Three Host Fruits

**Figure 1:**
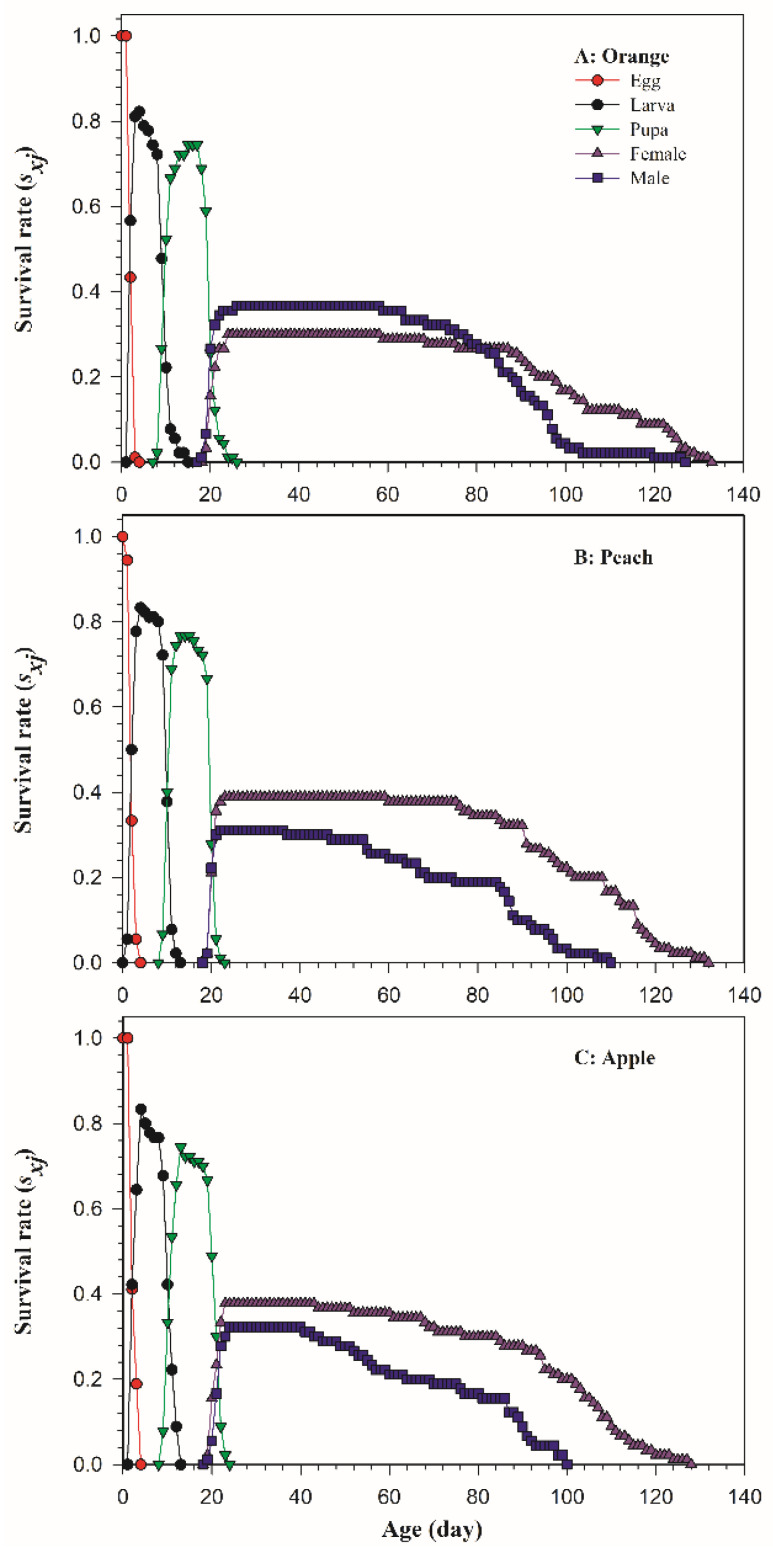
The age-stage specific survival rates (*s_xj_*) of *Bactrocera dorsalis* on three host fruits: (**A**) orange; (**B**) peach; (**C**) apple.

The age-stage specific survival rates (*s_xj_*) of *B. dorsalis* on three host fruits are shown in Figure 1, and the curves show the survival probability from egg to age *x* and stage *j*. According to the analysis, the eclosion time of males and females on oranges was the earliest, on the 18th and 17th day, respectively (Figure 1A), followed by on peaches and apples, both the 19th day (Figure 1B,C). The curves of different instar stages overlapped, which was caused by the different growth and developmental rates of *B. dorsalis* individuals.

**Figure 2:**
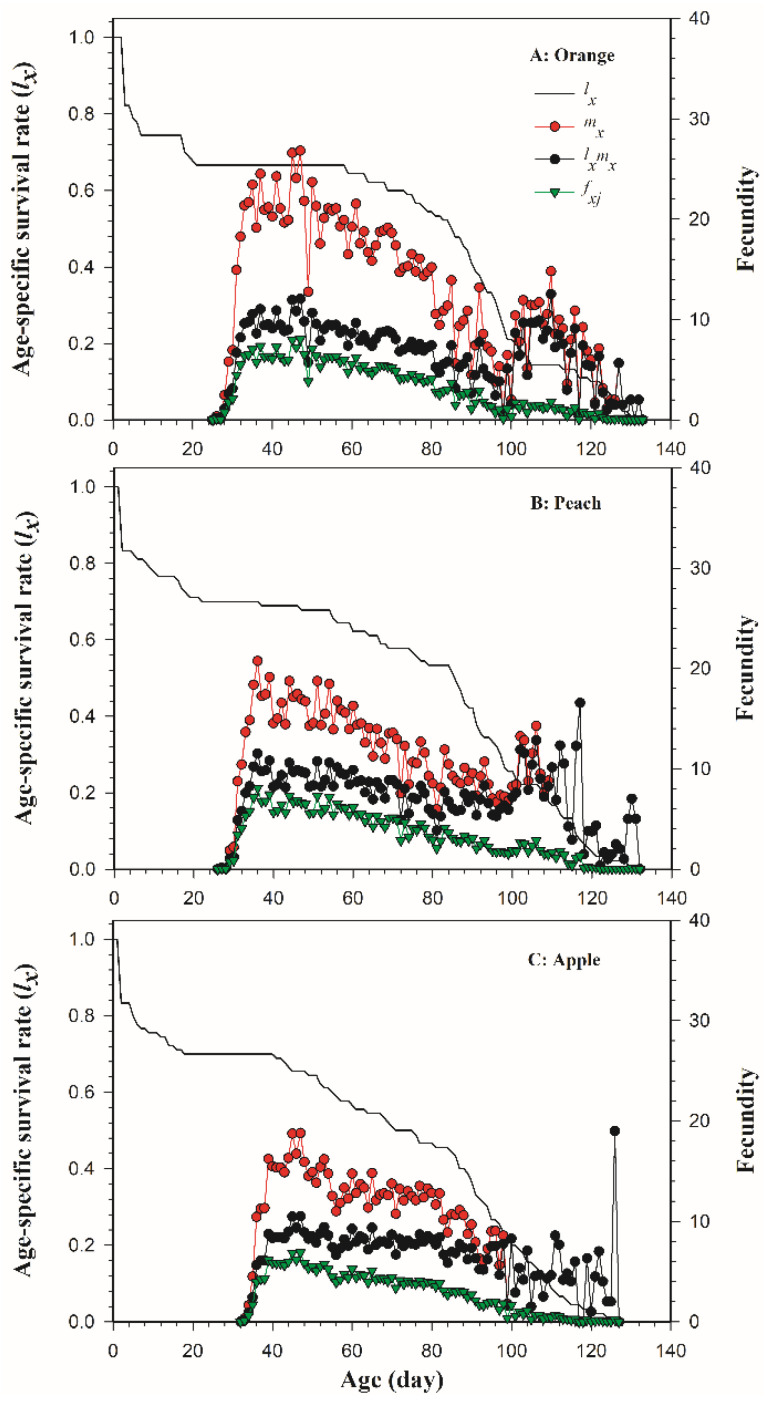
The age-specific survival rate (*l_x_*), the age-stage specific fecundity (*f_xj_*), the age-specific fecundity (*m_x_*), and age-specific maternity (*l_x_m_x_*) of *Bactrocera dorsalis* on three host fruits: (**A**) orange; (**B**) peach; (**C**) apple.

The age-specific survival rate (*l_x_*), age-stage specific fecundity (*f_xj_*), age-specific fecundity (*m_x_*), and age-specific maternity (*l_x_m_x_*) of *B. dorsalis* on three host fruits are shown in Figure 2. It was found that the fecundity of *B. dorsalis* females appeared on the 26th day on oranges, earlier than on peaches (27th day) and apples (33th day). The overall age-specific maternity (*l_x_m_x_*) of *B. dorsalis* on oranges (Figure 2A), peaches (Figure 2B), and apples (Figure 2C) peaked on the 48th, 51th and 48th day, respectively, with 12.07, 10.59, and 10.51 eggs.

As shown in Table 3, the three fruit hosts had no significant difference on net reproductive rate (*R*_0_), which was the highest on peaches (354.11), followed by oranges (347.20) and apples (273.21). The intrinsic rate of increase (*r*) and finite rate of increase (*λ*) also showed no significant differences on oranges (0.1266 day^−1^ and 1.1349 day^−1^, respectively) and on peaches (0.1235 day^−1^ and 1.1314 day^−1^, respectively), though both were significantly higher than on apples (0.1077 day^−1^ and 1.1137 day^−1^; orange: *P* = 0.0014, *P* = 0.0014; peach: *P* = 0.0018, *P* = 0.0017). As for the mean generation time (*T*) of *B. dorsalis*, significant differences were shown on the three host fruits, with the longest on apples (52.08 day) followed by peaches (47.54 day, *P* < 0.0001), and the shortest on oranges (46.21 day, *P* = 0.0001).

**Figure 3:**
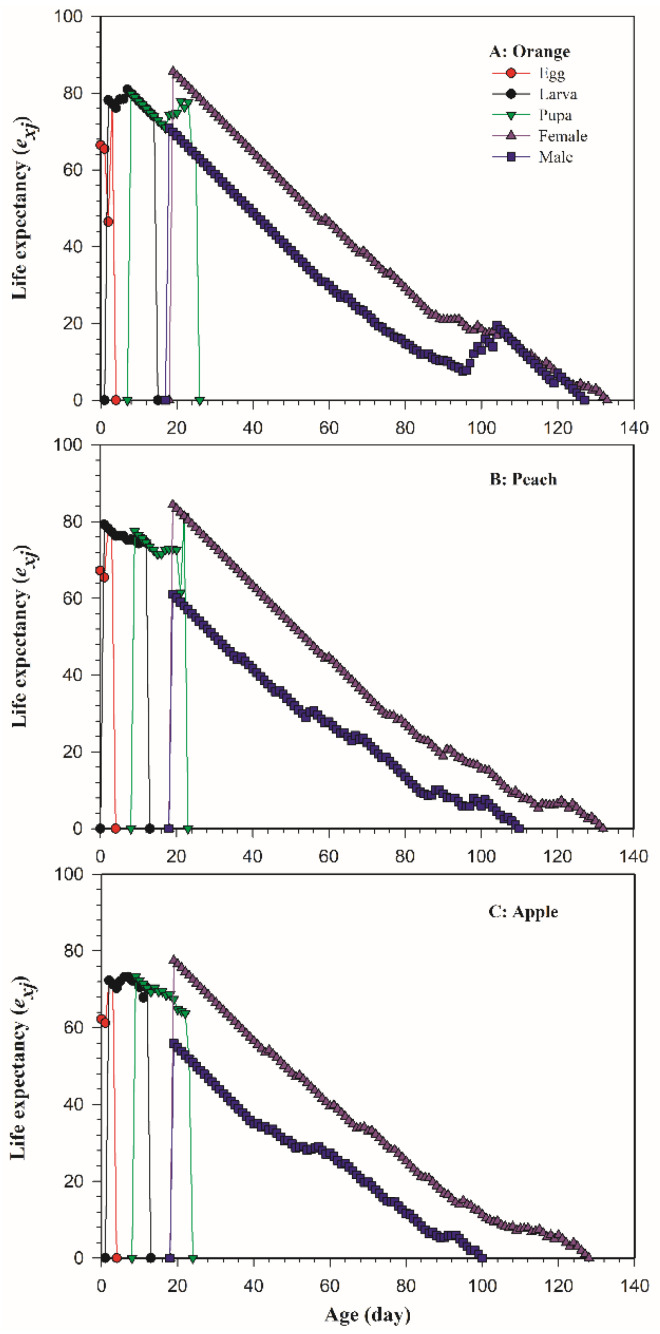
The age-stage life expectancy (*e_xj_*) of *Bactrocera dorsalis* on three host fruits: (**A**) orange; (**B**) peach; (**C**) apple.

Figure 3 shows the age-stage life expectancy (*e_xj_*) of *B. dorsalis* on different host fruits. This parameter can be used to predict the survival time of individuals in the future and plays an important role in assessing the damage degree and duration of individual pests. At age zero (*e*_01_), the life expectancy of *B. dorsalis* on oranges (Figure 3A), peaches (Figure 3B), and apples (Figure 3C) was 66.46 day, 67.21 day, and 62.24 day, respectively, which is completely consistent with the total longevity in Table 2.

**Figure 4:**
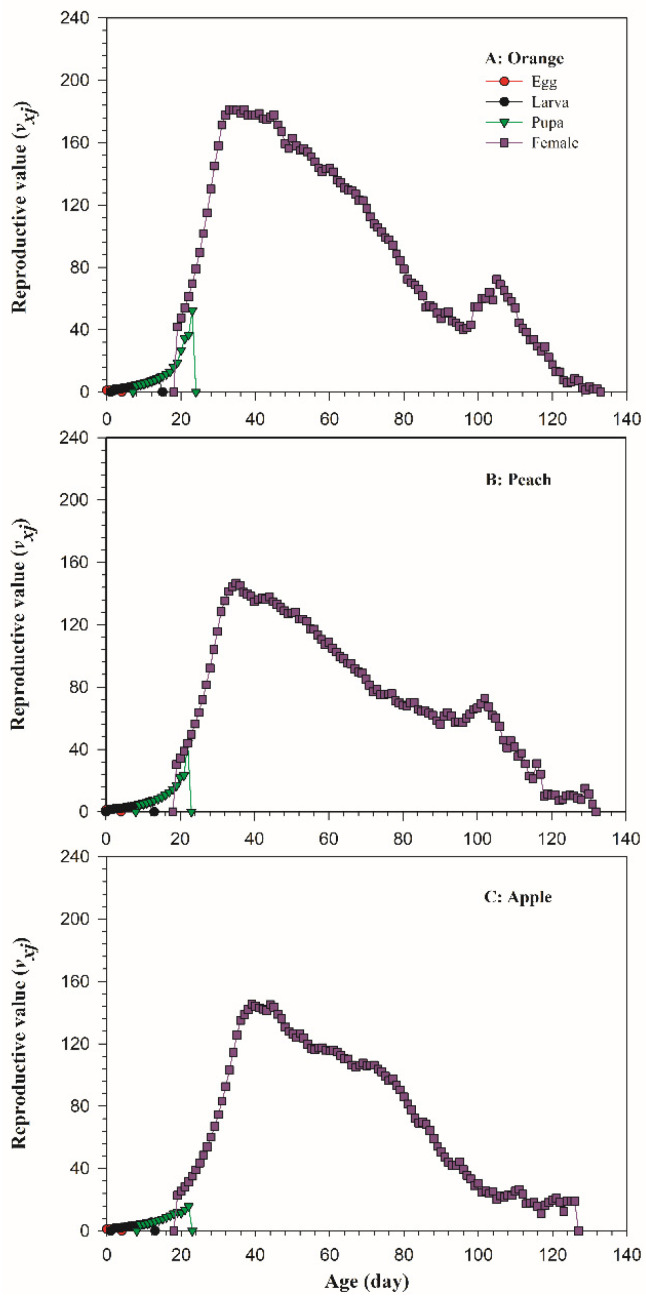
The reproductive value (*v_xj_*) of *Bactrocera dorsalis* on three host fruits: (**A**) orange; (**B**) peach; (**C**) apple.

The reproductive value (*v_xj_*) refers to the contribution of individual pests at age *x* and stage *j* to the future population growth, and can be used to assess the rate of population growth. At age zero (*v*_01_), the reproductive value of *B. dorsalis* on oranges, peaches, and apples was 1.1349 day^−1^, 1.1314 day^−1^, and 1.1137 day^−1^, respectively, which is the same as the finite rate of increase (*λ*). The reproductive value curves showed peaks at 181.02 day^−1^ on 34 day for oranges (Figure 4A), 146.67 d^−1^ on 35 day for peaches (Figure 4B), and 145.47 day^−1^ on 39 day for apples (Figure 4C), with the highest and earliest occurrence on oranges, followed by peaches and apples.

### 3.3. Population Prediction of B. dorsalis on Three Host Fruits

**Figure 5:**
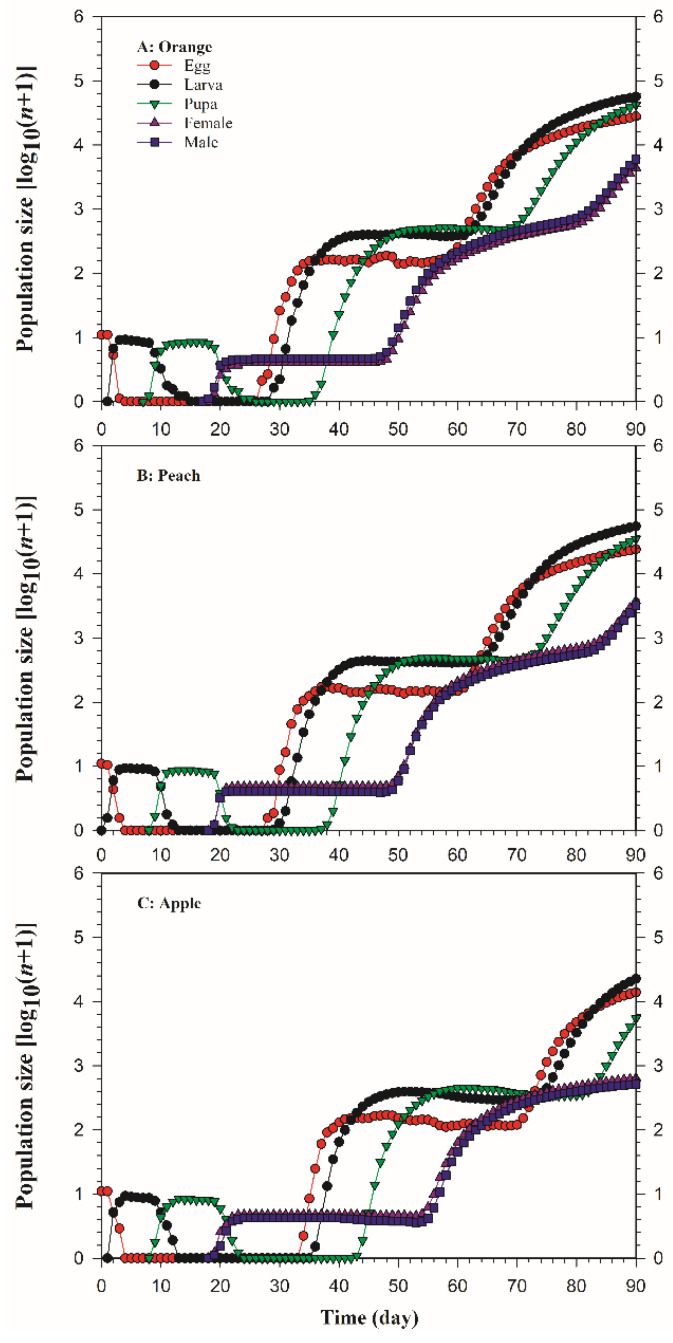
Population prediction of *Bactrocera dorsalis* on three host fruits. An initial population of 10 eggs was used in each projection: (**A**) orange; (**B**) peach; (**C**) apple.

**Figure 6:**
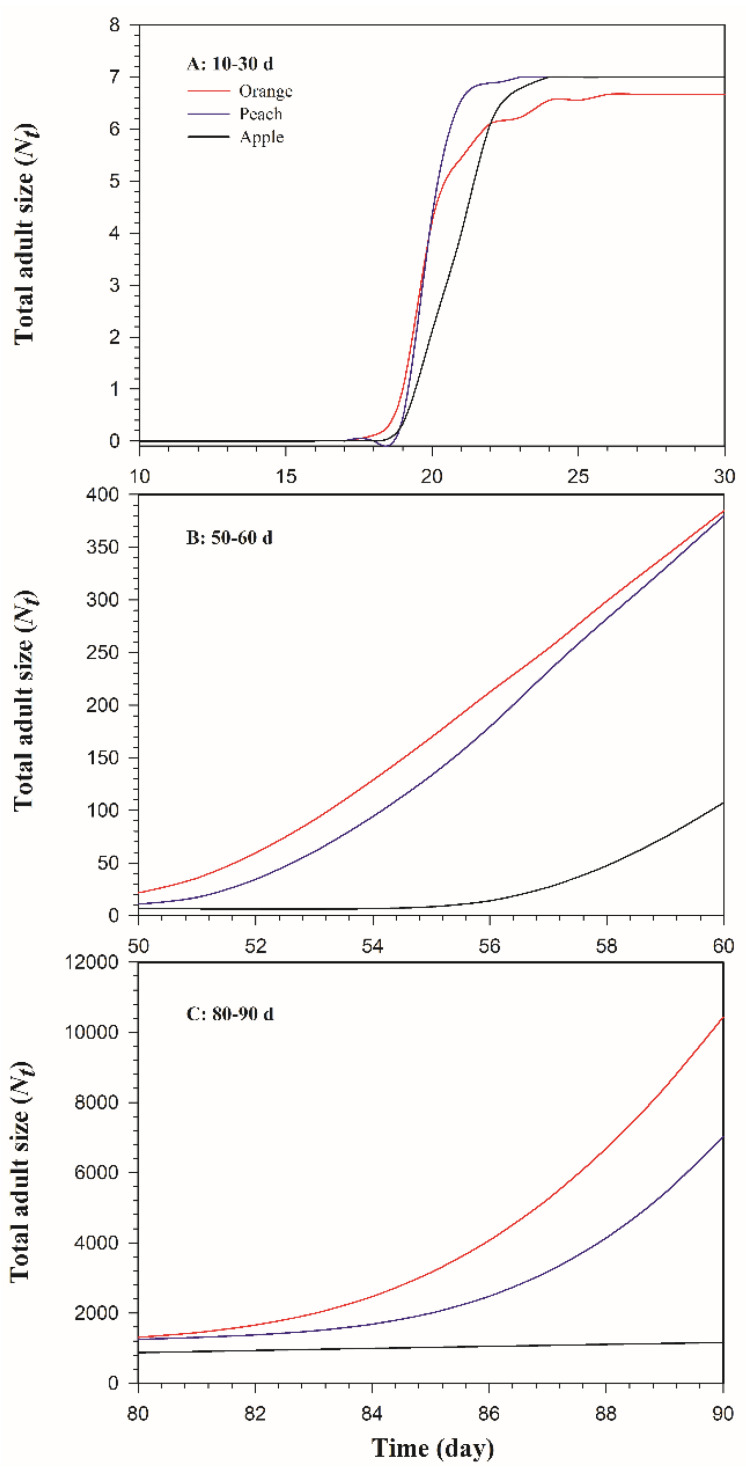
The total adult size (*N_t_*) of *Bactrocera dorsalis* on three host fruits in the following time intervals: (**A**) 10–30 d, (**B**) 50–60 d, (**C**) 80–90 d.

As shown in Figure 5, the TIMING-MSChart program was used to predict the population growth based on 10 eggs. The results showed that *B. dorsalis* populations reared on three different host fruits all had an obvious growth trend. *Bactrocera dorsalis* adults started to appear on the 17th day on oranges and on the 19th day on both peaches and apples (Figure 6A). The adult population growth was the fastest on oranges, with a total of 10,428 individuals, 90 days after the oviposition, followed by that on peaches, with a total of 7018, and the slowest on apples, which, at 1160, was only 11.12% of the adult population on oranges (Figure 6B,C).

## 4. Discussions

Host plants are an important factor affecting the survival, growth, development, and fecundity of herbivorous insects. Generally, if an insect possesses a short developmental time, high survival rate, and fecundity on a host plant, it indicates that the host plant is suitable for the insect [27]. With the further invasion of *B. dorsalis* into northern China, an age-stage two-sex life table was constructed to clarify whether *B. dorsalis* was causing damage to peach and apple trees in northern China. This method not only takes into account of male individuals, which are generally ignored in the traditional female age-specific life tables, but also fully considers the differences among individual insects and the death of some pre-adult individuals [14,15].

### 4.1. Potential Fitness of B. dorsalis to Peaches and Apples

The age-stage two-sex life table of the second generation of *B. dorsalis* fed with peaches and apples was constructed by using the southern fruit of oranges as the control. It was found that *B. dorsalis* could complete its growth, development, and reproduction on peaches and apples, which are two dominant northern fruits, and could cause potential damage with its apparent population growth. Generally, the duration of each age-stage (such as egg stage, larval stage, pupal stage, and adult stage) can be used to describe the developmental period of an insect [28]. A shorter developmental period and a faster developmental rate of an insect usually indicate a greater fitness to its host. The egg duration, TPOP, and mean generation time (*T*) of *B. dorsalis* showed no significant difference on peaches and oranges, showing that the growth and developmental rate of *B. dorsalis* on peaches in the north was basically the same as that on oranges in the south. Furthermore, the intrinsic rate of increase (*r*) and finite rate of increase (*λ*) represent the instantaneous growth rate and the total growth rate of the population within a certain time period, both of which are important parameters closely related to the population growth potential of the species [29]. In this study, *B. dorsalis* showed no significant difference in intrinsic rate of increase (*r*) and finite rate of increase (*λ*) on peaches and oranges, indicating that its population growth potential was equally high on northern peaches and southern oranges.

The TIMING-MSChart program has often been used for population prediction in studies of insect life tables, such as those of *B. tabaci* [18] and *Laodelphax striatellus* (Fallen) (Hemiptera: Delphacidae) [30]. This program was also used in this study to predict the population growth trend of *B. dorsalis* 90 days later based on 10 eggs. It was found that the whole population increased by 13,667.3 times on oranges and 12,112.1 times on peaches, indicating that *B. dorsalis* will potentially cause great damage to peaches in the north. The whole population of *B. dorsalis* on apples increased by 4311 times, which is significantly lower than on peaches and oranges; In addition, *B. dorsalis* fed on apples showed no significant difference in the rates of hatching, pupation, eclosion, and pre-adult survival with those on oranges and peaches, illustrating that *B. dorsalis* still implies a great risk of damage on apples in the north.

### 4.2. Fecundity as a Key Indicator of B. dorsalis Fitness to Its Hosts 

*Bactrocera dorsalis*, as an important pest of *Bactrocera*, usually prefers to lay eggs in soft skinned fruits [31]. The larvae hatching from eggs feed on the pulp inside the fruit, inducing the immature fruits to turn yellow and fall off [32]. Therefore, the parameters of pre-adult duration (especially larval duration) on different hosts are important indicators of the direct damage caused by the insect [33]. It was found that *B. dorsalis* had no significant difference in the rates of hatching, pupation, eclosion, and pre-adult survival on the three host fruits, indicating that the above three hosts have little effect on pre-adult duration including egg, larval, and pupal durations; accordingly, the southern and northern host fruits have little difference on their effect on the growth and development of *B. dorsalis* before adult duration.

However, the intrinsic rate of increase (*r*), finite rate of increase (*λ*), and mean generation time (*T*) of *B. dorsalis* fed on the three hosts in this study were significantly different, indicating different fitness of *B. dorsalis* to the three hosts. The main reason for this phenomenon lies in the fact that the oviposition of *B. dorsalis* adults on oranges and peaches appears earlier and the peak value is higher. *B. dorsalis* also had longer oviposition days, higher reproductive value, and heavier pupal weight on oranges and peaches. All these showed that reproductive ability (especially fecundity) accounts for the different fitness of *B. dorsalis* to its hosts.

It has been reported that some nutrients, toxins, latex, and resin in the fruit restrain the growth, development and survival of insects [31]. For example, the phenol in the resin in immature fruits of Anacardiaceae plants suppressed the survival of immature *B. dorsalis* [34]. Based on the analysis of the pre-adult duration index of *B. dorsalis*, it was speculated that peach and apple fruits contained no substances inhibiting its development. Wang et al. [35] found that the number of eggs laid by *Grapholitha molesta* (Busck) (Lepidoptera: Tortricidae) was directly proportional to the sugar content of apples; accordingly, it is speculated the reason why the growth rate of *B. dorsalis* on apples was less than on oranges and peaches possibly lies in the fact that apples contain less nutrition favorable for *B. dorsalis* reproduction or contain a small number of substances that inhibits its reproductive ability. However, the specific effects on *B. dorsalis* of the substances in northern fruits and vegetables are still not clear, and further studies including composition determination of fruit substances, feeding tests, and molecular research are necessary.

### 4.3. Selected Parameters Taken into Consideration in the Use of Life Table Data

The life table data is a large data set. When applying the parameters longevity and oviposition days, the following two points should be considered carefully to ensure the statistics assessment is accurate and reliable.

#### 4.3.1. Longevity of Female and Male Adults Separately

The expression of adult duration is often used in statistics of adult development history, and many scholars use this term to describe the developmental period of adults [36]. However, this cannot reflect the real situation of male and female populations, since the adult durations of females and males are often different. As shown in this study, the longevity of *B. dorsalis* female adults was significantly longer than that of male adults on the same host fruit. Therefore, the longevity of female and male adults should also be counted separately to ensure data reliability. The same situation was also found in *B. tabaci* [18] and *L. striatellus* [30].

#### 4.3.2. Differentiating Oviposition Days and Oviposition Period

The fecundity of insects is closely related to the number of oviposition days. It is essential to distinguish oviposition days and oviposition duration in assessing the reproductive ability of the species, with the former referring to the actual oviposition days and the latter to the length of time from the first oviposition day to the last day. When insects do not oviposit on a certain day or several days during the oviposition period, the oviposition days are inconsistent with the oviposition period. Therefore, it is quite natural that the oviposition period is often longer than the oviposition days; that is to say, a longer oviposition period does not necessarily mean higher fecundity. Therefore, oviposition days rather than oviposition period should be taken into consideration to assess the life table parameters of insect populations.

The detailed parameters of *B. dorsalis* and its population prediction on three host fruits in this study confirmed that *B. dorsalis* showed relatively high fitness to peaches and apples, with highly similar fitness between peaches and oranges, indicating a positive factor for *B. dorsalis* dispersal to northern China and a great damage risk to northern fruits. Peaches, as one of the dominant northern fruits, are most likely to become new potential host plants of *B. dorsalis* in north China. Therefore, the monitoring of *B. dorsalis* in northern China should be strengthened and further study is necessary to explore whether *B. dorsalis* can overwinter in the North or if it is annually transferred to the northern areas within transported fruits, so as to elucidate the mechanism of its continuous proliferation in the past decades. This will lay a theoretical foundation for integrated pest management and precise control of *B. dorsalis* in northern orchards.

## Figures and Tables

**Table 1:** The hatching rate, pupation rate, eclosion rate, and pupal weight of *Bactrocera dorsalis* on three host fruits.

Statistics	Mean ± SE ^1^		
	Orange	Peach	Apple
Hatching rate (%)	82.22 ± 4.14 a	83.33 ± 0.00 a	83.33 ± 4.60 a
Pupation rate (%)	90.22 ± 2.95 a	92.00 ± 2.62 a	89.29 ± 4.48 a
Eclosion rate (%)	90.78 ± 3.31 a	91.67 ± 4.36 a	93.89 ± 3.68 a
Pupal weight (mg)	16.33 ± 0.25 a	15.06 ± 0.19 b	14.38 ± 0.22 c

^1^ Significant differences between different treatments of the same parameter are indicated by a, b, c (*p* < 0.05).

**Table 2:** Means and standard errors of the developmental durations, pre-adult survival rate, longevity, fecundity, TPOP, female proportion in cohort (*N_f_*/*N*), and male proportion in cohort (*N_m_*/*N*) of *Bactrocera dorsalis* on three host fruits.

Statistics	Orange	Peach	Apple
Egg duration (d)	2.32 ± 0.06 b	2.40 ± 0.08 b	2.72 ± 0.09 a
Larval duration (d)	7.84 ± 0.15 a	8.13 ± 0.08 a	8.12 ± 0.11 a
Pupal duration (d)	10.62 ± 0.18 a	9.90 ± 0.08 b	10.30 ± 0.06 a
Pre-adult survival rate (%)	66.67 ± 4.97 a	70.01 ± 4.84 a	70.03 ± 4.83 a
Adult duration (d)	75.42 ± 2.33 a	72.63 ± 2.67 ab	65.35 ± 2.86 b
Female adult longevity (d)	83.78 ± 3.70 aA	82.89 ± 2.84 aA	75.53 ± 3.45 aA
Male adult longevity (d)	68.58 ± 2.42 aB	59.82 ± 3.63 bB	53.41 ± 3.65 cB
Total longevity (d)	66.46 ± 4.69 a	67.21 ± 4.58 a	62.24 ± 4.40 a
Oviposition days (d)	66.00 ± 3.43 a	63.86 ± 2.88 a	52.50 ± 3.00 b
Oviposition period (d)	73.48 ± 4.01 a	71.26 ± 3.13 a	59.35 ± 3.48 b
Fecundity (F) (eggs)	1157.33 ± 54.07 a	910.57 ± 49.19 b	723.21 ± 46.57 c
TPOP (d)	29.30 ± 0.33 c	30.14 ± 0.26 b	35.85 ± 0.35 a
Proportion of female adult (N*_f_*/*N*)	0.30 ± 0.05 a	0.39 ± 0.05 a	0.38 ± 0.05 a
Proportion of male adult (N*_m_*/*N*)	0.37 ± 0.05 a	0.31 ± 0.05 a	0.32 ± 0.05 a
*N*, *N_f_*	90, 27	90, 35	90, 34

Standard errors were estimated using 100,000 bootstrap resampling. The paired bootstrap test was used to detect the differences between different hosts. Significant differences between different treatments of the same parameter are indicated by a, b, c. Significant differences between different parameters of the same treatment are indicated by A and B (*p* < 0.05).

**Table 3:** Means and standard errors of the net reproductive rate (*R*_0_), intrinsic rate of increase (*r*), finite rate of increase (*λ*), and mean generation time (*T*) of *Bactrocera dorsalis* on three host fruits.

Statistics	Mean ± SE ^1^		
	Orange	Peach	Apple
*R*_0_ (offspring/individual)	347.20 ± 58.24 a	354.11 ± 50.45 a	273.21 ± 40.90 a
*r* (d^−1^)	0.1266 ± 0.0044 a	0.1235 ± 0.0035 a	0.1077 ± 0.0033 b
*λ* (d^−1^)	1.1349 ± 0.0050 a	1.1314 ± 0.0039 a	1.1137 ± 0.0036 b
*T* (d)	46.21 ± 0.48 c	47.54 ± 0.44 b	52.08 ± 0.62 a

^1^ Standard error was estimated using 100,000 bootstrap resampling. The paired bootstrap test was used to detect the differences between different hosts. Significant differences between different treatments of the same parameter are indicated by a, b, c (*p* < 0.05).

## Data Availability

The data presented in this study are available on request from the corresponding author.

## Supplementary Materials

The following supporting information can be downloaded at: https://www.mdpi.com/article/10.3390/insects13030258/s1, Figure S1: Damage of *Bactrocera dorsalis* on three host fruits.
