# Supplementary material for: Use of Age-Stage, Two-Sex Life Table to Compare the Fitness of Bactrocera dorsalis (Diptera: Tephritidae) on Northern and Southern Host Fruits in China
Source: Insects. 2022 Mar 4;13(3):258. doi: 10.3390/insects13030258 (PMC8954565; doi:10.3390/insects13030258)
Supplement: Supplementary file 1 [file insects-13-00258-s001.zip › insects-1562378-supplementary.pdf]

insects

MDPI

Supplementary Materials

Use of Age-Stage, Two-Sex Life Table to Compare the Fitness of *Bactrocera dorsalis* (Diptera: Tephritidae) on Northern and Southern Host Fruits in China

Yanfei Zhu, Fangjian Qi, Xiumei Tan, Tong Zhang, Ziwen Teng, Yinjun Fan, Fanghao Wan and Hongxu Zhou

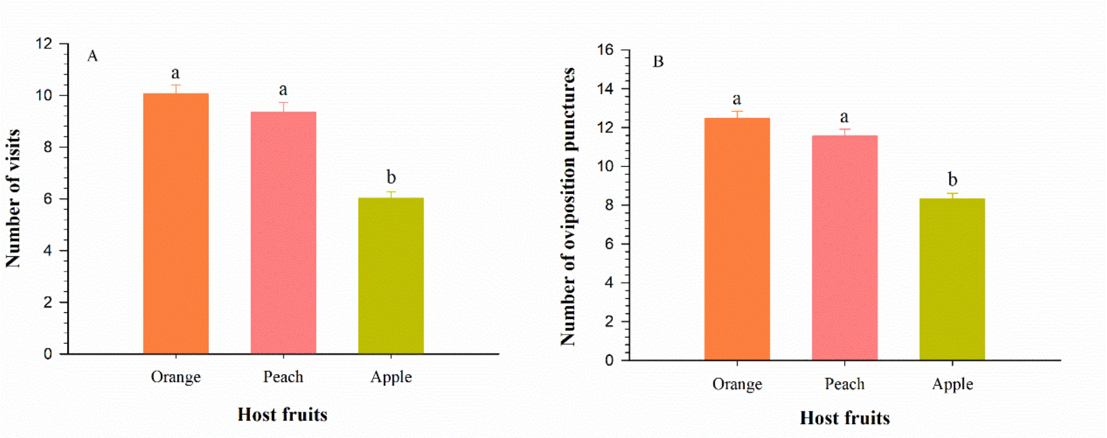

Figure S1. Damage of *Bactrocera dorsalis* on three host fruits. (A) Number of visits; (B) Number of oviposition punctures. Experimental methods and data processing reference. In Figure S1, the experimental and data processing methods were all referred to Jaleel et al., 2018.

References

1. Jaleel, W.; Lu, L.H.; He, Y.R. Biology, taxonomy, and IPM strategies of *Bactrocera tau* Walker and complex species (Diptera: Tephritidae) in Asia: a comprehensive review. *Environ. Sci. Pollut. Res.* **2018**, *25*, 19346–19361.
